# Shear models and parametric analysis of the PVC geomembrane-cushion interface in a high rock-fill dam

**DOI:** 10.1371/journal.pone.0245245

**Published:** 2021-01-22

**Authors:** Yun-Feng Liu, Ke Gu, Yi-Ming Shu, Xian-Lei Zhang, Xin-Xin Liu, Wen-long Mao

**Affiliations:** 1 College of Water Conservancy and Hydropower Engineering, Hohai University, Nanjing, P. R. China; 2 School of Water Conservancy, North China University of Water Resources and Electric Power, Zhengzhou, P. R. China; 3 School of Human Habitation and Environment, Nanchang Institute of Science and Technology, Nanchang, P. R. China; China University of Mining and Technology, CHINA

## Abstract

As a type of flexible impermeable material, a PVC geomembrane must be cooperatively used with cushion materials. The contact interface between a PVC geomembrane and cushion easily loses stability. In this present paper, we analyzed the shear models and parameters of the interface to study the stability. Two different cushion materials were used: the common extrusion sidewall and non-fines concrete. To simulate real working conditions, flexible silicone cushions were added under the loading plates to simulate hydraulic pressure loading, and the loading effect of flexible silicone cushions was demonstrated by measuring the actual contact areas under different normal pressures between the geomembrane and cushion using the thin-film pressure sensor. According to elastomer shear stress, there are two main types of shear stress between the PVC geomembrane and the cushion: viscous shear stress and hysteresis shear stress. The viscous shear stress between the geomembrane and the cement grout was measured using a dry, smooth concrete sample, then the precise formula parameters of the viscous shear stress and viscous friction coefficient were obtained. The hysteresis shear stress between the geomembrane and the cushion was calculated by subtracting the viscous shear stress from the total shear stress. The formula parameters of the hysteresis shear stress and hysteresis friction coefficient were calculated. The three-dimensional box-counting dimensions of the cushion surface were calculated, and the formula parameters of the hysteresis friction were positively correlated with the three-dimensional box dimensions.

## 1 Introduction

As a type of flexible impermeable material, a PVC geomembrane must form an impermeable structure with cushion materials to play an impermeable role. The shear strength between the interface of the PVC geomembrane and cushion material directly affects the stability, security, and work quality of the impermeable structure of the geomembrane, especially in faced rock-fill dams. Many laboratory tests and site tests have shown that the shear stress-shear displacement relationship of different geosynthetic and cushion material interfaces present different features, and researchers have proposed various types of constitutive models for description based on these characters.

Girard et al. analyzed the instability of the interface between the geotechnical cloth and the geomembrane in an embankment project. Through analysis, they found that the main cause of this event was overestimation of the interfacial shear strength [1]. Wu et al. investigated the failure of a geosynthetic lining reservoir. The results showed that the initial design error and improper selection of geomembranes were the main causes of failure [2]. Dixon et al. proposed the corresponding design methods and parameter acquisition methods for construction design through the anti-shear test of geosynthetics [3]. However, Dixon et al. noted that the corresponding calculation formula for geosynthetics obtained through experiments did not fully coincide with the actual project, and they suggested that the design guidance should be proposed by performing and summing error analyses of the test data [4]. Sia and Dixon found that the interfacial shear strength conformed to the normal distribution [5]. Seed et al., Byrne, Shallenberger, and Filz, Jones, Dixon, and Gomez and Filz noted that the strength of the synthetic interface of most geosynthetics was susceptible to the strain-softening behavior, suggesting that the peak strength is significantly greater than the residual strength of geosynthetics [6–10].

To analyze the security and stability of real projects, Giroud and Beech as well as Koerner et al. proposed the limit equilibrium method (LEM) [11, 12]. Filz et al. analyzed the progressive failure process of the geosynthetic interface in landfills using the finite element method. The numerically calculated results showed that progressive failure may significantly affect the stability of urban solid waste landfills [13]. Ge et al. Carried out the interval analysis of dam break loss and established the economic risk criteria of the dam [14, 15].

Wu proposed the mechanical models of different shear stages by analyzing the characteristics of the geosynthetic interfacial shear and suggested using different shear strengths in different areas of a project through the LEM and flac3d simulation [16].

The common calculation method is to conduct the fitting process of the interfacial shear stress-shear displacement relationship curve. Then, the actual projects are analyzed by numerical simulation. However, the fitted constitutive equation does not explain the essence of the geomembrane shearing phenomenon in principle. Based on the elastomer friction, this paper analyzed the components and characteristics of the geomembrane shearing phenomenon.

## 2 Separation of hysteresis friction and viscous friction

### 2.1 Elastomer friction theory

PVC geomembranes are flaky membranes made by extruding PVC plastic particles after adding modifiers. The physical and mechanical properties of PVC geomembranes vary with different formulations. When the PVC geomembranes in this paper were at 20°C, their ultimate strain was approximately 250%. The storage modulus was approximately 15 MPa, and the loss modulus was approximately 3.5 MPa. The stress-strain relationship showed obvious viscoelastic characteristics. These features indicate that the modified PVC geomembrane is an elastomeric material. Elastomers are a type of viscoelastic polymer. Compared to other materials, elastomers generally have a lower Young modulus and higher failure strain [17].

Elastomeric materials have obviously different mechanical and frictional properties from traditional materials. The normal pressure affects the actual contact area between elastomers and rough contact surfaces, and when the normal pressure on elastomers increases, the actual contact area between elastomers and rough contact surfaces increases, and the frictional force increases accordingly. However, the inherent characteristics of elastomers will increase the frictional force less than the increase in normal pressure, which causes a decrease in the friction coefficient. When elastomers are in full contact with the micro-bulges on contact surfaces, the actual contact area between elastomers and rough contact surfaces no longer increases, and the friction coefficient between elastomers and rough contact surfaces tends to stabilize.

**Fig 1:**
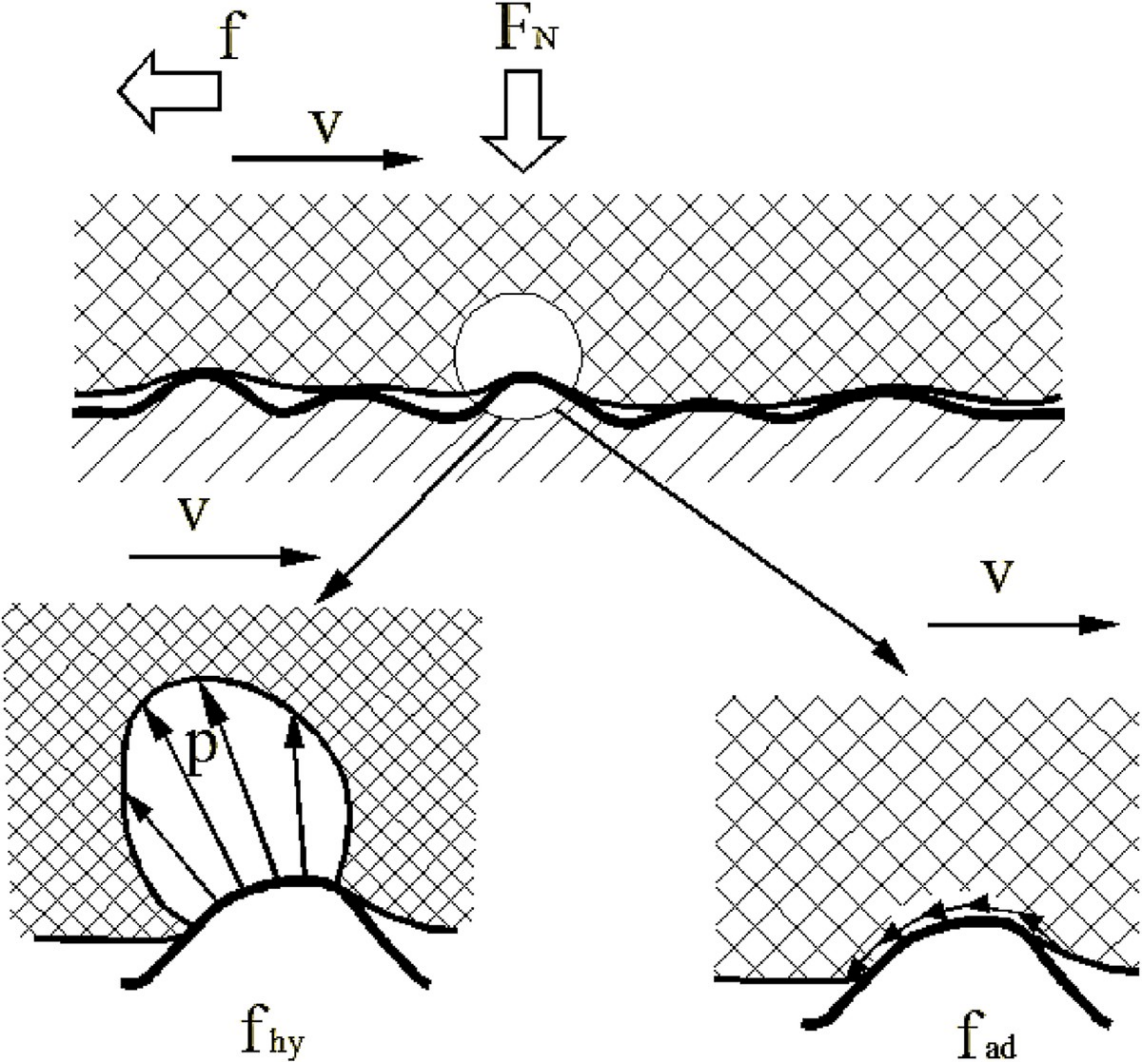
Diagram of the frictions between a PVC geomembrane and rough surface.

The friction between elastomers and a rough surface includes viscous friction, hysteresis friction, mechanical friction, fluid viscous friction, and the resistance caused by surface tension effects. Although friction contains many factors, elastomer friction mainly consists of two major parts, hysteresis friction and viscous friction. Fig 1 shows a diagram of viscous and hysteresis friction. As shown in Fig 1, when the PVC geomembrane is in contact with a rough surface, the PVC geomembrane will be deformed in the bulging part of the rough surface due to the low elastic modulus, while a part of the energy in the PVC geomembrane will be dissipated. Because of the horizontal velocity, the stress distribution in the contact area is not eudipleural. There is a component in the horizontal direction, which causes the hysteresis shear stress. Meanwhile, in the contact layer between the PVC geomembrane and rough surface, because the PVC geomembrane has a lower elastic modulus, its contact area with the rough surface is much larger than the contact area between conventional materials. At this time, the molecular van der Waals force has a non-negligible effect and constitutes the viscous shear stress.

Where F_N_ is the normal pressure, f is the horizontal force, v is the horizontal velocity, f_hy_ is hysteresis friction, f_ad_ is the viscous friction, and p is the stress distribution.

### 2.2 Friction separation technical route

The two most important components in the friction of elastic materials are the viscous components and hysteresis components, which can be separated by free adjustment of the test conditions to determine the value of each component. There are two common methods: using lubricants to minimize or eliminate the viscous component and using smooth, dry surfaces to eliminate the hysteresis component [18]. In this study, the method eliminating the hysteresis component was adopted.

**Fig 2:**
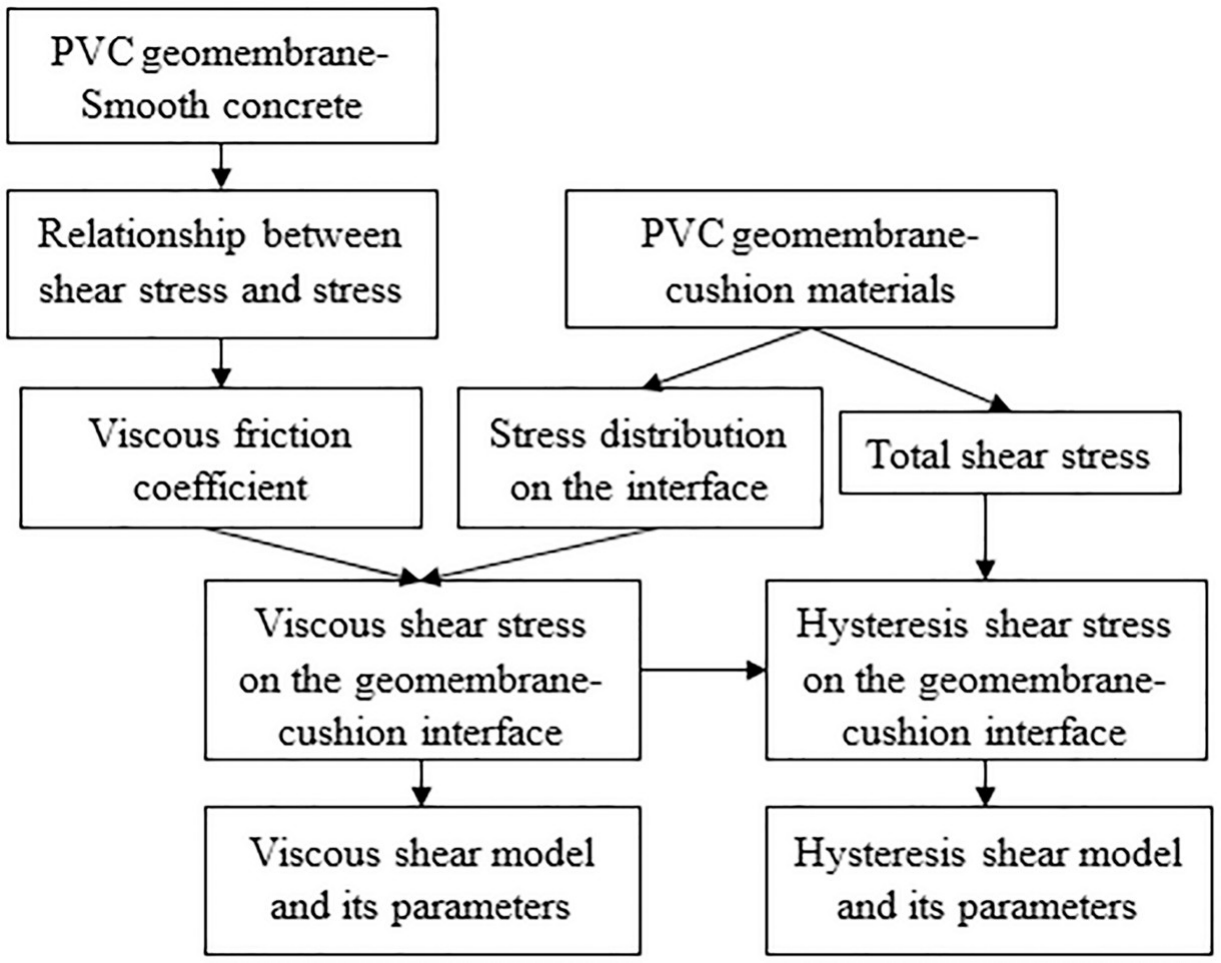
Friction separation technical route.

Fig 2 shows a diagram of the friction separation technical route. Using a smooth, dry concrete sample, the relationship between the viscous friction and its coefficient with the normal stress was solved by measuring the viscous shear stresses under different normal stresses. Then, according to the stress distribution on the geomembrane and cushion, the viscous friction under different conditions can be calculated. According to the total shear stress measured by the direct shear apparatus, the hysteresis shear stress and its coefficient were worked out by combining the viscous friction values. The hysteresis friction is equal to the difference between the total friction and viscous friction.

### 2.3 Samples and direct shear apparatus

At present, there are two types of commonly used cushion materials in projects: non-fines concrete and extrusion sidewall. The correlation coefficients and proportions are shown in Table 1. The largest difference between non-fines concrete and extrusion sidewall is in the use of sand materials. Considering the uncertainty of the source of non-fines concrete aggregate in actual projects, crushed stone aggregates were adopted in this paper. The particle size range of non-fines concrete sample aggregates was 5–10 mm. Mechanical jolt ramming for 30 s after the sample was poured was performed to ensure the evenness of the sample surface. The measured porosity rate of the non-fines concrete sample was 20%.

**Table 1:** Proportions of concrete components.

Cushion	Cement	Sand	Gravel	Water/cement ratio	Porosity
Non-fines concrete	378 kg/m^3^	0 kg/m^3^	1536 kg/m^3^	0.299	20%
Extrusion sidewall	80 kg/m^3^	651 kg/m^3^	1449 kg/m^3^	1.31	0%
Smooth concrete	290 kg/m^3^	805 kg/m^3^	984 kg/m^3^	0.55	0%

**Fig 3:**
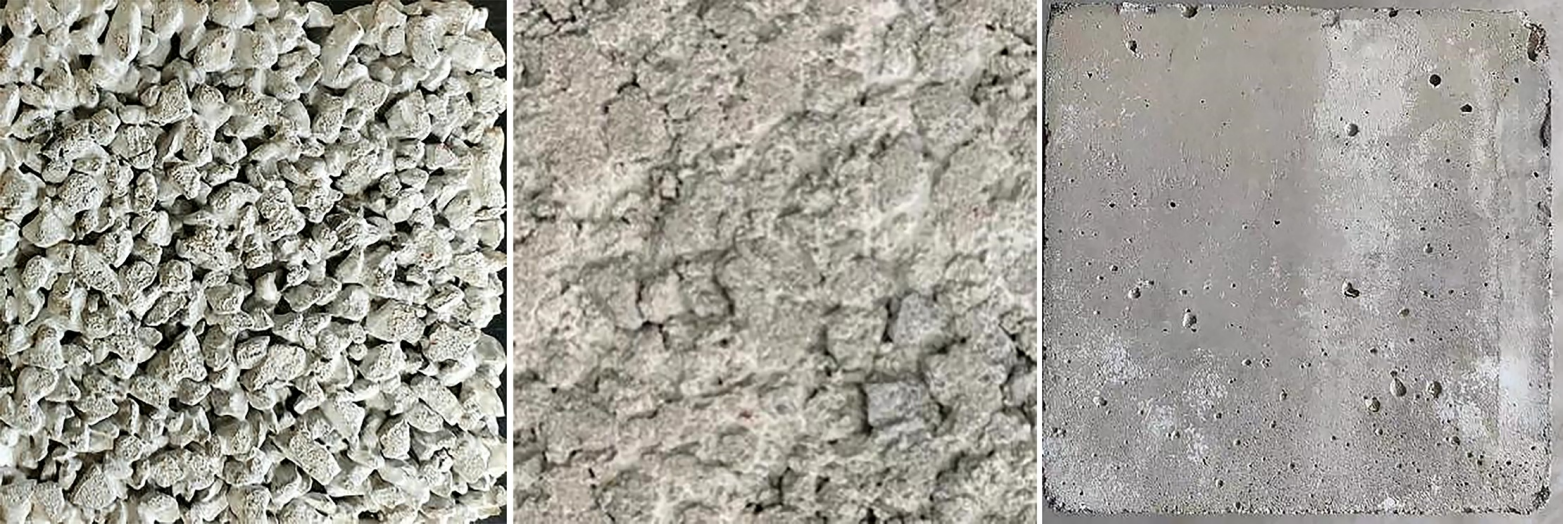
Different cushions. (a) Non-fines concrete. (b) Extrusion sidewall. (c) Smooth concrete.

Fig 3 shows real images of the 5 to 10 mm particle-size non-fines concrete, extrusion sidewall, and smooth concrete. There are obvious depressions on the surface of the non-fines concrete sample, the surface of the extrusion sidewall is relatively flat, with no large depressions, and the smooth concrete surface is completely smooth. Thus, there are obvious differences in the sensitivity of different cushion materials to normal stress.

PVC geomembrane used is specially produced by a Chinese company for test. The materials are rolled. The geomembrane roll is 2m in wide and 2mm in thick.

TGH-2C geosynthetics direct shear test system is used in the direct shear test. The interface normal load and horizontal shear load are provided by weight lever system and motor respectively. The maximum effective size of the upper and lower shear boxes is 300 × 300 mm. During the test, the upper shear box is fixed and the lower shear box moves to produce shear effect. The tests applied 7 pressure values (35, 50, 100, 150, 200, 250, and 300 kPa) in the normal direction, and the shear rate was set to 1 mm/min. The tests were carried out in a relative humidity of 60±10% and a temperature of 21±2°C according to the ASTM standard.

## 3 Simulated hydraulic pressure loading shear test

### 3.1 Silicone to simulate hydraulic pressure loading

Common geotechnical shear test materials are earth materials and geosynthetics, and the normal forces are loaded with loading plates. Earth material is low in strength, and under small normal stress, it deforms and achieves full interfacial contact.

**Fig 4:**
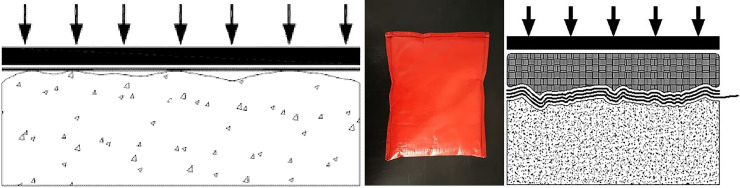
Diagram of the shear test loading improvement. (a) The conventional loading device. (b) The conventional loading device. (c) The test device.

The cushion material in this paper was rigid concrete. The non-fines concrete surface had large depressions, and while the surface of the extrusion sidewall was flatter, depressions remained. The conventional loading device is shown in Fig 4(A). From top to bottom are the loading plate, geomembrane, and cushion. Under the normal pressure, neither the PVC geomembrane nor the non-fines concrete showed large deformations, resulting in only partial interfacial contact. In actual projects, the geomembrane under the action of hydraulic pressure shows large deformations. The geomembrane under high hydraulic pressure will completely fit the non-fines concrete cushion. To simulate the actual project situation, based on the conventional loading device, the liquid silicone bag cushion in Fig 4(B) was added to simulate the hydraulic pressure. The test device is shown in Fig 4(C). From top to bottom are the loading plate, silicone cushion, geomembrane, and cushion. The device realized the simulated hydraulic loading condition.

### 3.2 Simulated hydraulic pressure effect

**Fig 5:**
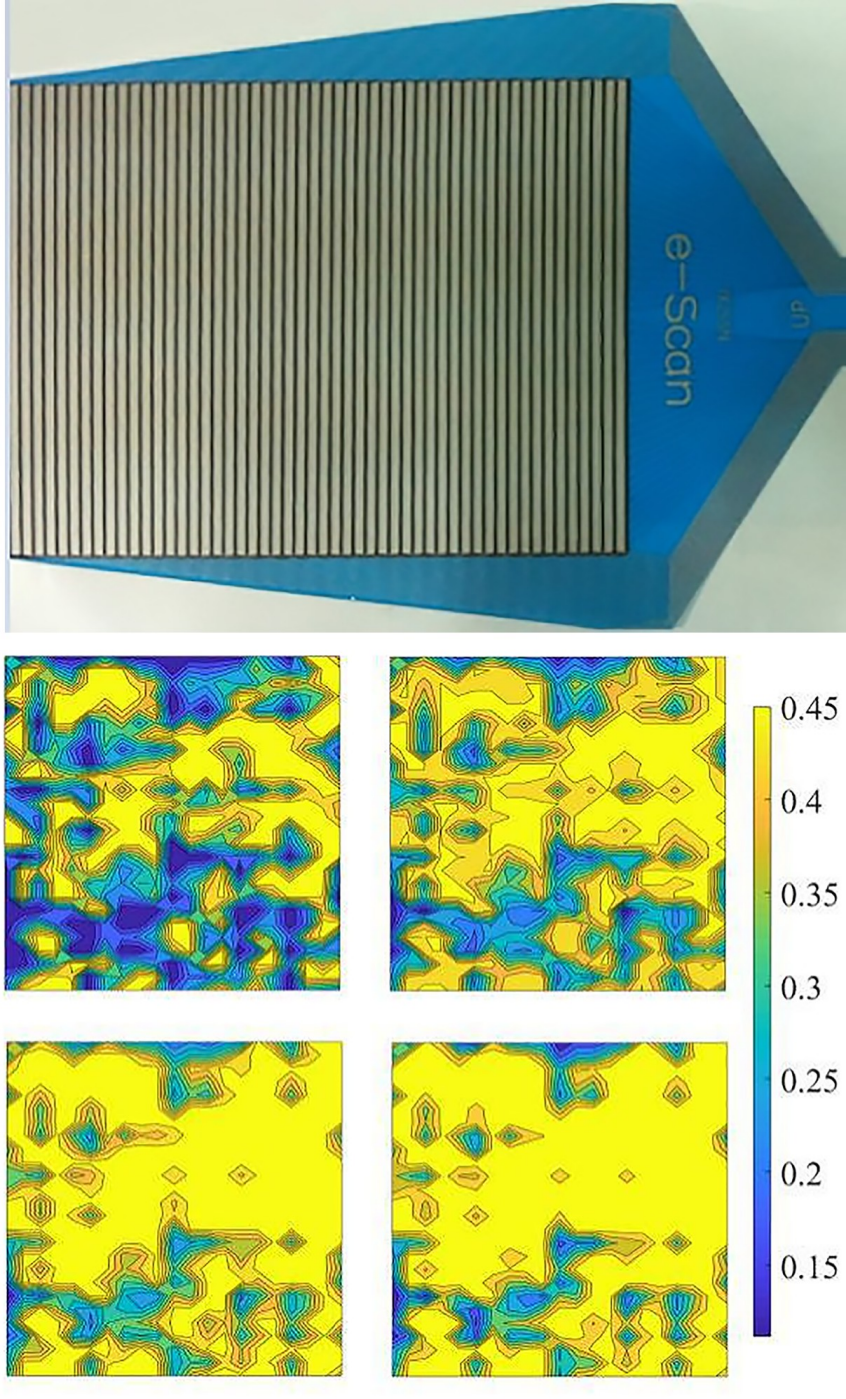
Stress distribution on the geomembrane-cushion contact area below 150, 200, 250, and 300 kPa. (a) The thin-film pressure sensor. (b) The stress distributions on the contact surface.

The thin-film pressure sensor is a new type of plane stress measurement instrument that can directly measure the stress on the sensor surface. The thin-film pressure sensor used had an induction area of 255 mm x 210 mm and an induction accuracy of 95%, as shown in Fig 5(A).

To test the effect of the silicone cushion in simulating hydraulic pressure loading, the actual contact areas under different normal pressures between the geomembrane and cushion were measured by the thin-film pressure sensor before the shear test to demonstrate the loading effect of the silicone material. The measurement arrangement of the contact surface stress is shown in Fig 5 from top to bottom are the loading plate, ultra-soft silicone, geomembrane, thin-film sensor, and non-fines concrete cushion.

Under a normal pressure of 150, 200, 250, and 300 kPa, the proportion of the contact area between the geomembrane and the non-fines concrete test block in the actual area of the cushion was 73.39%, 84.67%, 93.61%, and 97.68%, respectively. The data are shown in Table 2; the measured data show that after the silicone cushion was applied, under a normal pressure of 300 kPa, the cushion showed almost complete interfacial contact, and the silicone cushion simulated the hydraulic pressure loading well.

**Table 2:** Normal pressures and contact area proportions.

Normal stress (kPa)	150	200	250	300
Contact ratio (%)	73.39	84.67	93.61	97.68

Fig 5(B) shows the stress distributions on the contact surface between the geomembrane and the non-fines concrete test block. The unit of measure in this figure is MPa. Here, the yellow areas represent high stress, and the high-stress probabilities correspond to the rightmost probability values.

The area of high stress increases with normal pressure.

### 3.3 Conventional/Simulated hydraulic pressure loading shear data

**Fig 6:**
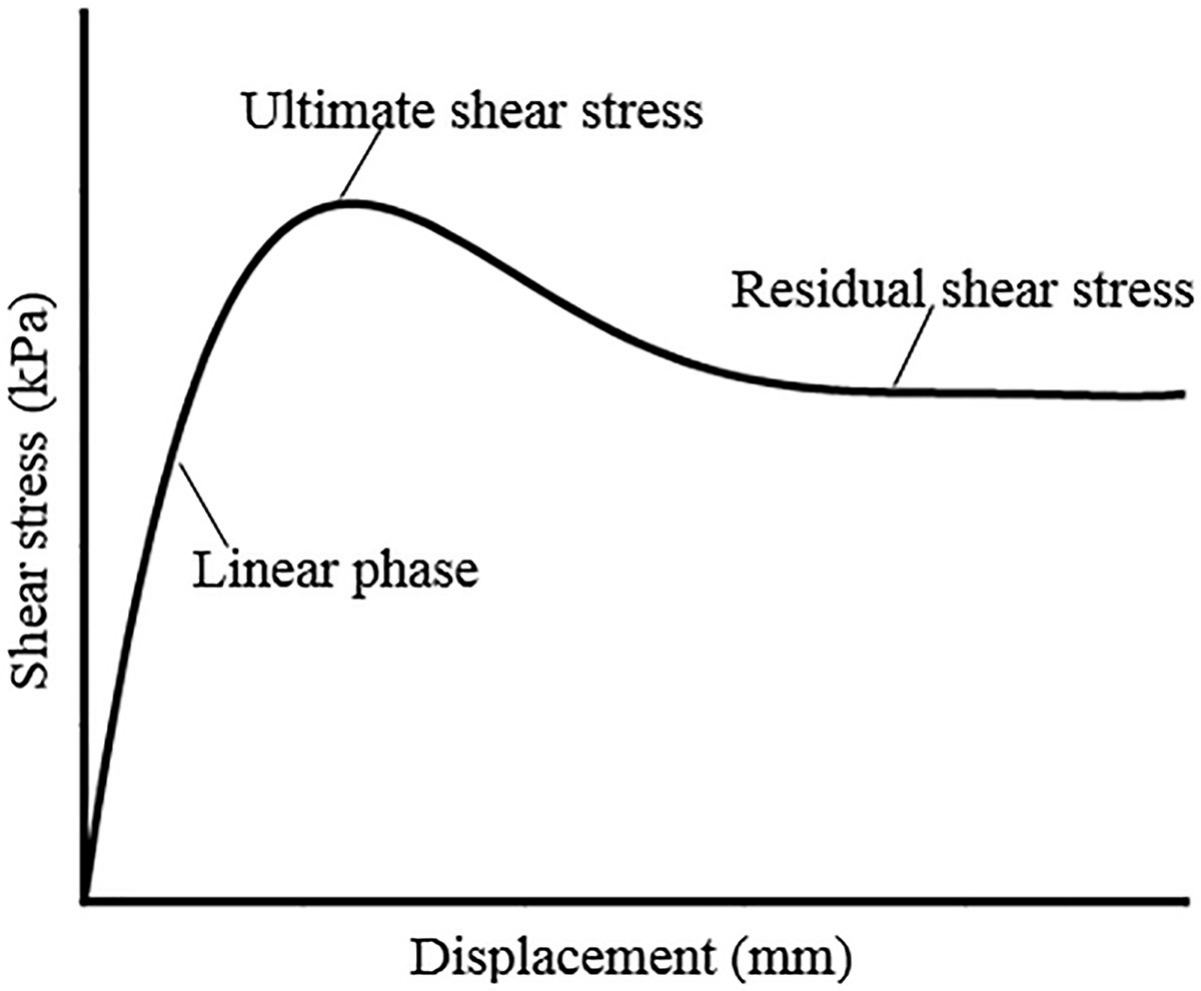
Diagram of the shear displacement and shear stress for the PVC geomembrane and non-fines concrete.

Fig 6 shows the typical shear displacement-shear stress relationship curve of the PVC geomembrane and extrusion sidewall/non-fines concrete cushions. Fig 6 shows a significant nonlinear relationship between the shear displacement and shear stress of the PVC geomembrane and extrusion sidewall/non-fines concrete before the ultimate shear stress. The shear stress no longer increases with the increase in shear displacement when the shear stress reaches the limit.

Measured shear stress data from the direct shear test of the PVC geomembrane and extrusion sidewall after the conventional shearing of 10 mm under normal stresses of 35, 50, 100, 150, 200, 250, and 300 kPa are shown in Table 3.

**Table 3:** Shear stresses of the extrusion sidewall.

Normal stress (kPa)	35	50	100	150	200	250	300
Shear stress (kPa)	9.78	13.52	26.56	39.64	52.75	65.84	78.73

Measured conventional shearing data from the direct shear test of the PVC geomembrane and non-fines concrete under normal stresses of 35, 50, 100, 150, 200, 250, and 300 kPa are shown in Table 4.

**Table 4:** Shear stresses of 5- to 10-mm particle-size non-fines concrete.

Normal stress (kPa)	35	50	100	150	200	250	300
Shear stress (kPa)	10.68	14.64	27.92	41.66	55.57	68.53	81.57

Measured shear stress data from the direct shear test of the PVC geomembrane and extrusion sidewall under simulated hydraulic pressure loading with normal stresses of 35, 50, 100, 150, 200, 250, and 300 kPa are shown in Table 5.

**Table 5:** Simulated hydraulic pressure loading shear stresses of the extrusion sidewall.

Normal stress (kPa)	35	50	100	150	200	250	300
Shear stress (kPa)	12.1	16.97	32.82	48.32	63.64	78.77	93.59

Measured shear stress data from the direct shear test of the PVC geomembrane and 5- to 10-mm particle-size non-fines concrete under simulated hydraulic pressure loading with normal stresses of 35, 50, 100, 150, 200, 250, and 300 kPa are shown in Table 6.

**Table 6:** Simulated hydraulic pressure loading shear stresses of 5- to 10-mm particle-size non-fines concrete.

Normal stress (kPa)	35	50	100	150	200	250	300
Shear stress (kPa)	15.3	20.55	38.14	55.37	71.78	87.57	104.12

**Fig 7:**
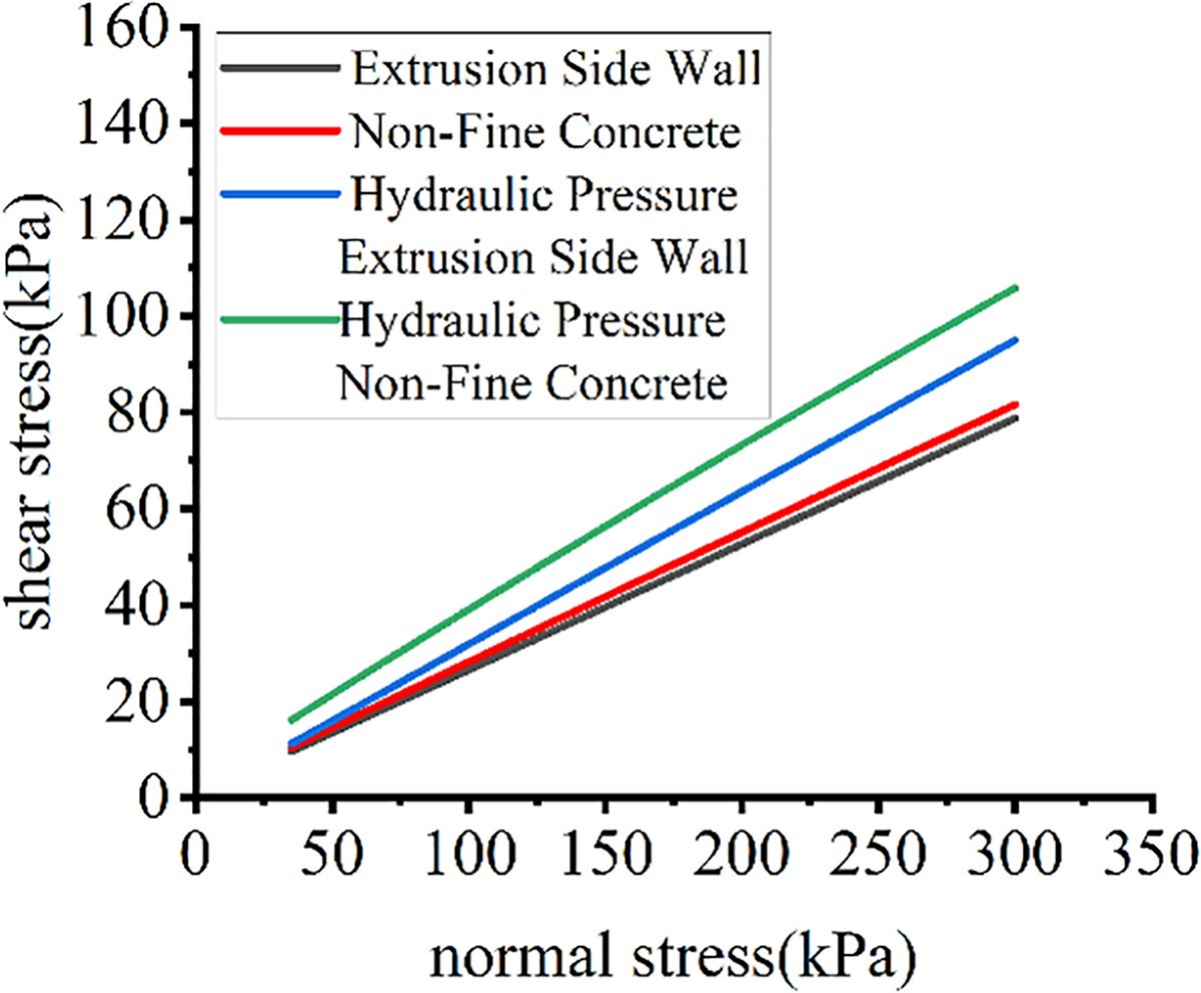
Test curves for different loading systems.

The normal stress-shear stress test curves for different cushions and loading forms are shown in Fig 7.

As shown in Fig 7, the silicone loading system simulating hydraulic pressure had a greater impact on shear stress; under conventional loading, there was no large difference in shear stress between different cushions. With the use of silicone to simulate hydraulic pressure loading, the difference in shear stress between different cushions increased. The interfacial contact area was different among different loading modes, and the hysteresis shear stress was obviously different. The measurement values of different test methods greatly varied. The conventional direct shear method had obviously small measurement values, and the silicone loading system simulating hydraulic pressure had more accurate measurement results.

## 4 Shear models of PVC geomembrane-cushion interfaces

### 4.1 Viscous shear model and its parameters

As shown in Fig 3, for both 5 to 10 mm particle-size non-fines concrete and extrusion sidewall concrete, all sample surfaces were wrapped in cement grout, and the contact materials were PVC plastics and cement grout between the interface of the PVC geomembrane and extrusion sidewall/non-fines concrete. To measure the relationship between viscous shear stress and normal stress, the shear test was performed using a smooth, dry concrete sample with the same cement grout, and the dry, smooth concrete sample is shown in Fig 3.

First, the viscous shear stresses under different normal stresses of the PVC geomembrane and dry, smooth concrete samples were measured by the geosynthetic direct shear apparatus.

The viscous shear stresses of the dry, smooth concrete test block under different normal stresses are shown in Table 7.

**Table 7:** Shear stresses of dry, smooth concrete under different normal stresses.

Normal stress (kPa)	35	50	100	150	200	250	300
Viscous shear stresses (kPa)	8.58	12.26	24.51	36.77	49.02	61.31	73.53

The elastomer is elastically deformed under pressure P. The viscous area is directly proportional to the normal stress and inversely proportional to the hardness [19]. Therefore, the relationship of the contact area with the normal stress and hardness is as follows:

$$A=K_{1}{\left(\frac{P}{E}\right)}^{n_{1}}$$ (1)

where A is the actual contact area, K_1_ is a constant, P is the normal stress, E is the storage modulus, and n_1_ is the material characteristics parameter. P/E is also known as the deformation degree factor; n1 is often slightly less than 1 [19].

The viscous shear stress F_A_ is the macro embodiment of the molecular van der Waals force, which is directly proportional to the actual contact area [19]. Therefore, the relationship between the viscous shear stress F_A_ and the contact area A is as follows:

$$F_{A}=K_{2}A=K{\left(\frac{P}{E}\right)}^{n_{1}}$$ (2)

where F_A_ is the viscous shear stress, K and K_2_ are constants, P is the normal stress, and E is the storage modulus.

The test data in Table 7 are the measured viscous shear stresses between the PVC geomembrane and cement grout. The data in Table 7 were fitted by Formula 2, and the fitted formula is as follows:

$$F_{A}=3.479\times {\left(\frac{P}{12.31}\right)}^{0.95}$$ (3)

where K is 3.479, and n_1_ is 0.95.

A more comprehensive study of adhesion shows that the precise formula for the viscous friction coefficient of elastomers and smooth surfaces is as follows [19]:

$$\mu_{A}=B\Phi (E/P^{1‐n_{1}})\tan\delta$$ (4)

where B is a constant, Ф is the interface shape function, E is the storage modulus, P is the normal stress, n_1_ is the material characteristic parameter, and tanδ is the loss tangent. The storage modulus E and loss tangent tanδ can be measured by thermodynamic analysis instruments.

The thermodynamic analysis test showed that the storage modulus E of the PVC geomembrane is 12.31 MPa, and the loss tangent tanδ is 0.2531. The viscous friction coefficients in Table 7 were fitted with Formula 4 and the measured parameters. The fitted formula is as follows:

$$\mu_{A}=0.1\times (12.31/P^{0.05})\times 0.2531$$ (5)

The 5 to 10mm particle-size non-fines concrete sample adopted in this paper showed a large degree of depression, and the contact area was not constant under different normal pressures. To accurately calculate the viscous shear stresses of the sample and PVC geomembrane under different normal stresses, the stress distribution under different normal stresses was first measured by the thin-film pressure sensor. Then, using the relationship between the viscous friction coefficient and pressure in Formula 5, the corresponding viscous friction coefficients under different normal stresses were calculated. With the distribution of normal pressure on the contact surface of the PVC geomembrane and non-fines concrete, the viscous frictional forces of each area under different normal pressures can be calculated, and the overall shear stress can be calculated by summing. The specific formula is as follows:

$$F_{A}=\sum_{i=1}^{n}P_{\left(i\right)}\mu_{A}(P_{\left(i\right)})$$ (6)

where P is the normal stress, and μ_A_ is the computational formula for the viscous shear stress.

The degree of depression of the extrusion sidewall was small. The data measured by the thin-film pressure sensor shows that under normal pressures of 150, 200, 250, and 300 kPa, full interfacial contact is achieved, so the viscous shear stress can be directly obtained by Formula 3.

The calculated viscous shear stresses are shown in Table 8.

**Table 8:** Viscous shear stresses under different normal stresses.

Normal stress (kPa)	35	50	100	150	200	250	300
Extrusion sidewall viscous shear stress (kPa)	9.39	13.17	25.45	37.40	49.15	60.85	72.25
Non-fines concrete viscous shear stress (kPa)	8.18	11.61	23.05	34.84	47	58.69	71.15

### 4.2 Hysteresis shear model and its parameters

The hysteresis shear stress component is represented as follows [19]:

$$F_{H}=MJ$$ (7)

In the formula, M is the total number of macro contact positions or the total number of micro-bulges in contact, and J is the frictional resistance at each contact position.

For individual bulge forms of different shapes, the hysteresis friction coefficient formula is derived from the viscoelastic mechanical model [19]:

$$\mu_{H}=4C{\left(P/E\right)}^{n_{2}}\tan\delta$$ (8)

In the formula, C is a constant, P is the normal stress, E is the storage modulus, n_2_ is a constant, and tanδ is the loss tangent. C and n_2_ are related to the bulge forms.

**Fig 8:**
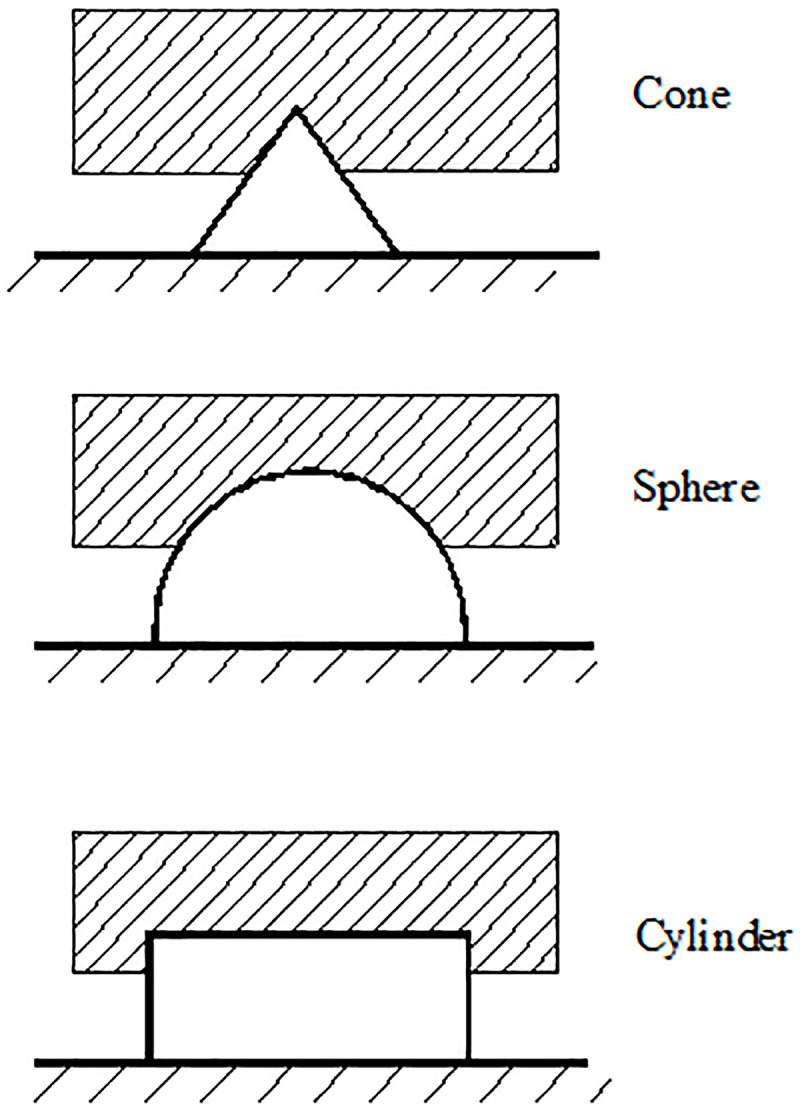
Diagram of hysteresis friction of different bulges.

For regular bulges in Fig 8, C and n_2_ can be determined by geometric calculations.

Overall bumps of the extrusion sidewall and non-fines concrete samples are not regular; the distribution of bumps on the whole sample surface is nonuniform. There are large bumps on the surface of the non-fines concrete, and there are micro-bumps on the surface of the large bumps. Therefore, parameters C and n_2_ in this paper are difficult to determine by geometric calculations.

To determine the hysteresis shear stresses under different normal stresses, the hysteresis shear stress F_H_ under different normal stresses can be obtained by calculating the viscous shear stress F_A_ of the extrusion sidewall and non-fines concrete samples to combine the total shear stress data in the direct shear test. The results of the calculations are shown in Table 9.

**Table 9:** Hysteresis shear stresses of the extrusion sidewall under different normal stresses.

Normal stress (kPa)	35	50	100	150	200	250	300
Extrusion sidewall hysteresis shear stress (kPa)	2.71	3.8	7.37	10.92	14.49	17.92	21.34
Non-fines concrete hysteresis shear stress (kPa)	7.12	8.94	15.09	20.53	24.78	28.88	32.97

By using Formula 8 for elastomer hysteresis shear stress and combining the hysteresis shear stress data in Tables 9 and 10, the hysteresis friction coefficient formula of different cushions can be calculated.

**Table 10:** Surface-fractal three-dimensional box dimensions of cushions.

	Smooth concrete	Extrusion sidewall	Non-fines concrete
Box dimensions	2	2.147	2.609

The hysteresis friction coefficient formula of the extrusion sidewall is as follows:

$$\mu_{H}=0.3173\times {\left(Ρ/12.31\right)}^{-0.0384}\times 0.0251$$ (9)

The hysteresis friction coefficient formula of 5 to 10 mm non-fines concrete is as follows:

$$\mu_{H}=1.065\times {\left(Ρ/12.31\right)}^{-0.279}\times 0.2531$$ (10)

**Fig 9:**
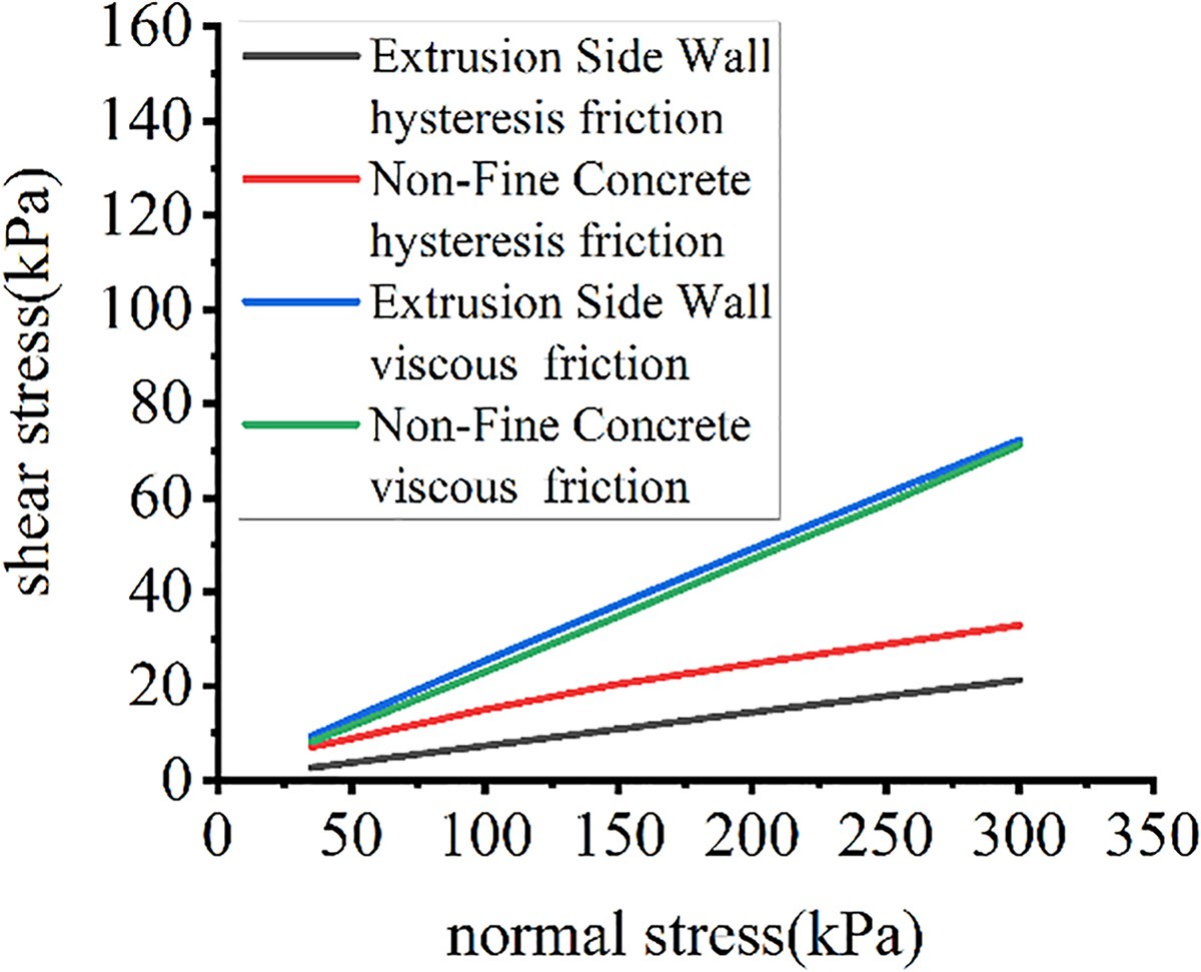
Distributions of each friction component.

The relationship of the normal pressure-shear stress component of different cushions is shown in Fig 9.

Both viscous and hysteresis shear stress are positively correlated with normal stress. The degree of depression on the surfaces of the cushion materials greatly affects the hysteresis shear stress.

The frictional force of the PVC geomembrane is related to the normal pressure, interfacial contact materials, and shape of the interface. The thin-film pressure sensor shows that the PVC geomembrane is in full contact with the non-fines concrete cushion under 300 kPa of normal stress. Therefore, with increasing normal pressure, the contact surface area no longer increases. Both viscous and hysteresis friction coefficients tend to be stable. Therefore, the frictional force under higher normal pressures can still be calculated by using the formula derived above.

### 4.3 Parameters of hysteretic friction coefficient and fractal 3D box-counting dimensions

The surface of the non-fines concrete has a complex and diverse irregular geometry, which is messy, discontinuous, and infinitely complex. However, at different scales, it geometrically repeats its irregular structures and has identical basic units, which is called self-similarity in fractal theory. The characteristic of having distribution characteristics independent of specific sizes is called scale invariance. Self-similarity and scale invariances are the essential features of fractal geometry and the main basis for establishing fractal measurement methods.

Fractal dimensions are calculated using many methods, such as Hausdorff dimensions, box dimensions, correlation dimensions, information dimensions, relevant dimensions, and packing dimensions. Box dimensions are also called box-counting dimensions, which are one of the most widely used dimensions. The primary algorithm of the three-dimensional box dimension algorithm involves covering the three-dimensional space on average by small cube boxes with a side length of L_s_ in the three-dimensional space and counting the number of boxes required to completely cover the graph N_s_. Then, by changing the side length of the cube, the number of boxes required to fully cover the graph is recounted. The three-dimensional box dimension can be calculated by Formula 11.

$$d_{s}=\frac{\ln N_{s}(L_{s})}{\ln L_{s}}$$ (11)

where d_s_ is the fractal constant, N_S_ is the number of boxes, and L_s_ is the size of the box.

In this paper, the three-dimensional data of the extrusion sidewall and the non-fines concrete cushions were obtained by three-dimensional scanning; then, the three-dimensional box dimensions were calculated by Formula 11.

The surface dimension calculations of the dry, smooth concrete, extrusion sidewall, and non-fines concrete are shown in Table 10.

Formula 8 shows that after the material of the contact surface has been determined, the hysteresis friction coefficient is only related to the cushion surface shape. The hysteresis friction coefficient parameters C and n_2_ of different cushion samples are shown in Table 11.

**Table 11:** Hysteresis friction formula parameters.

	Smooth concrete	Extrusion sidewall	Non-fines concrete
C	0	0.3173	1.065
n_2_	0	-0.0384	-0.279

**Fig 10:**
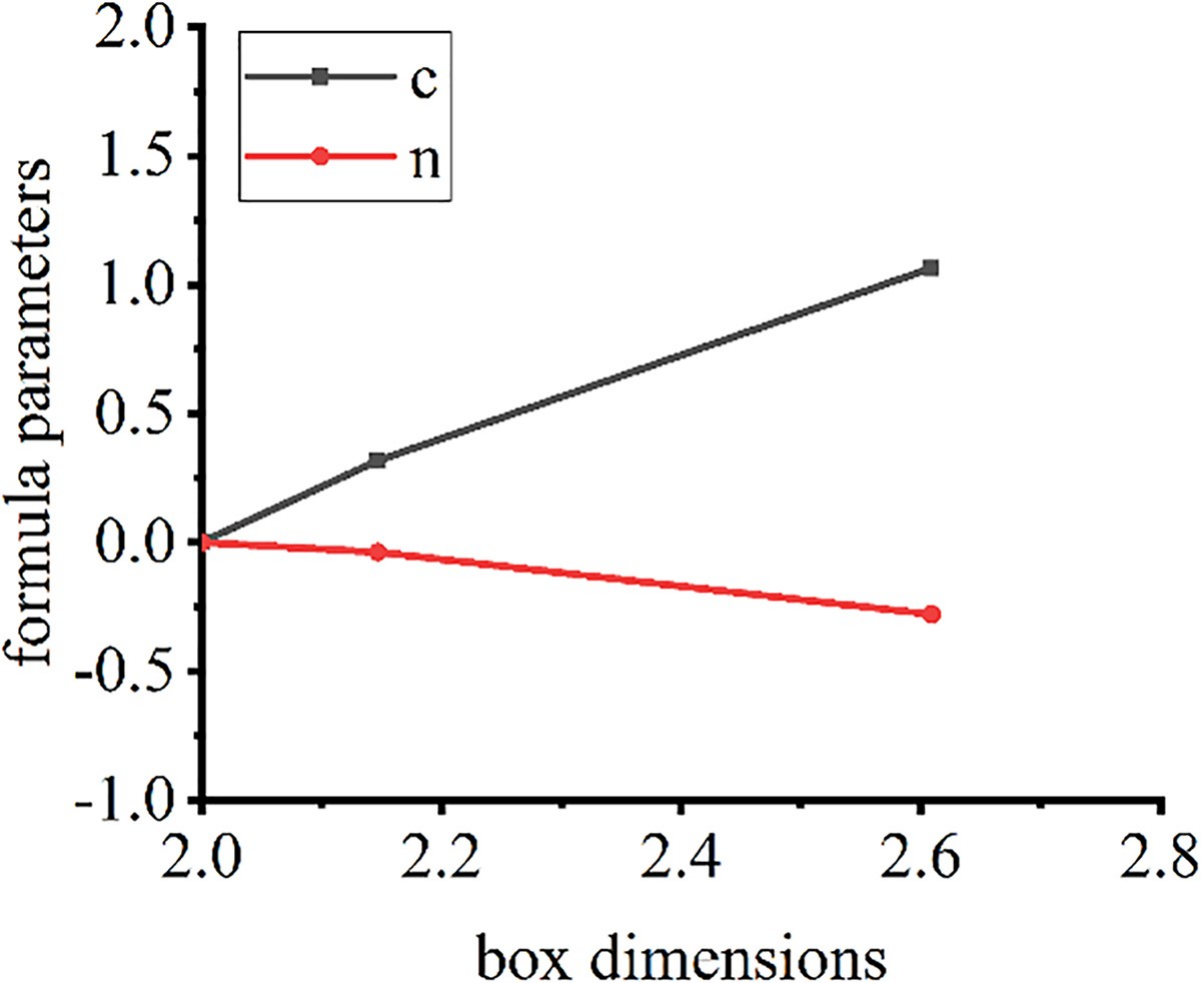
Fractal dimensions and hysteresis friction parameters.

The surface fractal dimensions and friction formula coefficient of the cushion samples are shown in Fig 10.

The surface fractal dimensions of dry, smooth concrete, extrusion sidewall, and non-fines concrete are basically linearly related to the parameters of the friction stress formula. For the new sample, the effect of its surface shape on the hysteresis frictional force can be determined by calculating the box dimensions through a three-dimensional analysis of its surface.

## 5 Conclusion

1. In this paper, common extrusion sidewall and non-fines concrete cushion materials were selected for the study of PVC geomembrane-cushion interface stability. Because conventional shear tests cannot well simulate the real hydraulic pressure, silicone was used to convert the normal pressure loaded by loading plates to a type of pressure similar to hydraulic pressure. The thin-film pressure sensor confirmed that the silicone cushion was significantly effective.
2. This work, through the elastomer theory, determined that there are mainly two types of shear stresses between the PVC geomembrane and the extrusion sidewall/non-fines concrete cushion: viscous stress and hysteresis stress. By referencing the elastomer friction theory and using the corresponding measurement methods, the viscous shear stress between the PVC geomembrane and the extrusion sidewall/non-fines concrete cushion was accurately measured, and the formula parameters of the elastomer viscous shear stress were determined. For contact interfaces of identical materials, this parameter has extensive practical value.
3. Under different normal stresses, through the total shear stress measured by test devices, the viscous shear stress can be obtained using the viscous shear stress formula. The difference between the two values is the hysteresis shear stress. Finally, the elastomer hysteresis shear stress formula parameters were determined.
4. Through the 3D box-counting dimensions that can be obtained by three-dimensional scanning data of the smooth, dry concrete, the extrusion sidewall, and the non-fines concrete, combined with the formula parameters of the three-dimensional box-counting dimensions and the hysteresis friction, we determined that the sample hysteresis model parameters are linearly correlated with the surface three-dimensional box-counting dimensions. For new cushion materials, by studying the surface dimensions of samples, we can measure the hysteresis friction effect to guide engineering design and construction.
